# Characterization of the PF-ILD phenotype in patients with advanced pulmonary sarcoidosis

**DOI:** 10.1186/s12931-022-02094-7

**Published:** 2022-06-25

**Authors:** M. C. Schimmelpennink, D. B. Meek, A. D. M. Vorselaars, L. C. M. Langezaal, C. H. M. van Moorsel, J. J. van der Vis, M. Veltkamp, J. C. Grutters

**Affiliations:** 1 Interstitial Lung Diseases Center of Excellence, Department of Pulmonology, St Antonius Hospital, Koekoekslaan 1, 3435 CM Nieuwegein, The Netherlands; 2 Interstitial Lung Diseases Center of Excellence, Department of Radiology, St Antonius Hospital, Nieuwegein, The Netherlands; 3 Department of Radiology, Treant Health Care Group, Emmen, Hoogeveen, Stadskanaal, The Netherlands; 4 Interstitial Lung Diseases Center of Excellence, Department of Clinical Chemistry, St Antonius Hospital, Nieuwegein, The Netherlands; 5 Division of Heart and Lungs, University Medical Center, Utrecht, The Netherlands

**Keywords:** PF-ILD, Advanced sarcoidosis, Mortality, Diffusion capacity

## Abstract

**Background:**

Advanced pulmonary sarcoidosis causes significant morbidity and can lead to death. Large trials demonstrated efficacy of antifibrotics in patients with progressive fibrosing interstitial lung diseases (PF-ILD), including a few with sarcoidosis. To date, little is known about this progressive fibrosing phenotype in sarcoidosis. Diffusion capacity of carbon monoxide (DLCO) may be a useful functional marker to screen for advanced pulmonary sarcoidosis. In this study, we describe a cohort with advanced pulmonary sarcoidosis and we gain insights in the progressive fibrosing phenotype in sarcoidosis.

**Methods:**

Patients with sarcoidosis and a DLCO < 50% predicted were included in this retrospective cohort study. First measurement of DLCO < 50% predicted was the baseline. Lung function data, HRCT, pulmonary hypertension (PH) and mortality were collected. Patients with > 10% fibrosis on HRCT meeting the criteria for ILD-progression within 24 months were labelled as PF-ILD. With Cox-regression analysis predictors of mortality were established.

**Results:**

106 patients with a DLCO < 50% predicted were included. Evolution of forced vital capacity (FVC) varied widely between patients from − 34% to + 45% after 2 years follow-up, whereas change in DLCO varied between − 11% and + 26%. Fourteen patients (15%) met the PF-ILD criteria, of whom 6 (43%) died within 10 years versus 10 (13%) in the non PF-ILD group (p = 0.006). PH was present 12 (11%), 56 (53%) demonstrated > 10% fibrosis on HRCT. Independent predictors of mortality and lung transplantation in the whole cohort are PH, PF-ILD and UIP-like pattern.

**Conclusion:**

In conclusion, within this group with advanced pulmonary sarcoidosis disease course varied widely from great functional improvement to death. PF-ILD patients had higher mortality rate than the mortality in the overall pulmonary sarcoidosis group. Future research should focus on the addition of antifibrotics in these patients.

*Trial registration* retrospectively registered

## Introduction

Sarcoidosis is a multi-organ granulomatous disorder characterized by a wide variety of clinical phenotypes [1]. About one third of patients with sarcoidosis develop a chronic course of disease [2]. Chronic pulmonary sarcoidosis can cause significant morbidity due to progressive fibrosis, pulmonary hypertension (PH), aspergilloma or other respiratory infections. Strikingly, several studies demonstrated that death rate of sarcoidosis has increased over the last decades [3, 4]. Respiratory insufficiency is the main cause of death of sarcoidosis in the western world [4, 5]. Advanced age, extensive fibrosis on high-resolution computed tomography (HRCT) and the presence of PH have been identified as predictors of mortality in sarcoidosis [6, 7].

Recently, the INBUILD trial revealed that nintedanib slowed disease progression in a heterogeneous group of patients with progressive fibrosing interstitial lung diseases (PF-ILD). The majority of patients consisted of patients with fibrotic hypersensitivity pneumonitis (fHP) and connective tissue disease (CTD)-ILD. This large randomized controlled trial also included several patients with sarcoidosis [8]. The outcomes of the INBUILD trial therefore suggests that treatment with antifibrotic therapy might potentially be useful in patients with sarcoidosis who meet the criteria for PF-ILD. However, nintedanib can cause significant side effects and is relatively expensive and therefore patients to be treated are ideally carefully selected. To date, it is unknown how many patients with sarcoidosis actually meet the criteria for PF-ILD.

The different clinical phenotypes in sarcoidosis make it difficult to predict its course. Walsh and colleagues developed a clinico-radiological risk-stratification system to identify patients with sarcoidosis at risk. This composite score included the following variables: CPI (composite physiological index), main pulmonary artery diameter to ascending aorta diameter ratio (MPAD/AAD ratio) and presence of more than 20% fibrosis on the HRCT [9]. As the authors mention in their paper, the prognostic strength of the CPI might be the incorporation of diffusion capacity (DLCO) to capture increased sensitivity to PH as well as the prognostic effect of DLCO in interstitial lung disease [9]. Given the somewhat laborious formula, the CPI is hardly used in daily practice whereas the DLCO is an integral part of pulmonary function testing in ILD. The combination of prognostic strength as well as overall availability should make the DLCO a useful tool in screening for advanced sarcoidosis [10]. DLCO negatively correlates with saturation during exercise in patients with ILD [11]. In addition, in patients with a diffusion capacity less than 50% a desaturation of 4% or more was found during exercise [11]. This change in saturation might have a great impact on experiencing dyspnea during exercise. For these reasons, we chose DLCO < 50% as entry criteria for selecting patients with advanced pulmonary sarcoidosis.

The aim of this paper is to describe functional and radiological characteristics and prognosis of a cohort with patients with advanced pulmonary sarcoidosis defined by severely limited DLCO. We investigated how many patients with advanced pulmonary sarcoidosis meet the criteria for PF-ILD and the prognosis of this subgroup.

## Methods

In this retrospective study cohort, we included 106 patients with sarcoidosis with at least at one time point a DLCOc of less than 50% of predicted. All patients with sarcoidosis with a DLCOc < 50% of predicted and with an HRCT at the baseline ± 1 year at St. Antonius Hospital Nieuwegein, the Netherlands between 1996 and 2018 were selected. The first time point at which the DLCOc was < 50% of predicted in our hospital since the diagnosis sarcoidosis was defined as baseline. Sarcoidosis was diagnosed according to the guidelines of ERS/ATS/WASOG [12].

**Fig. 1:**
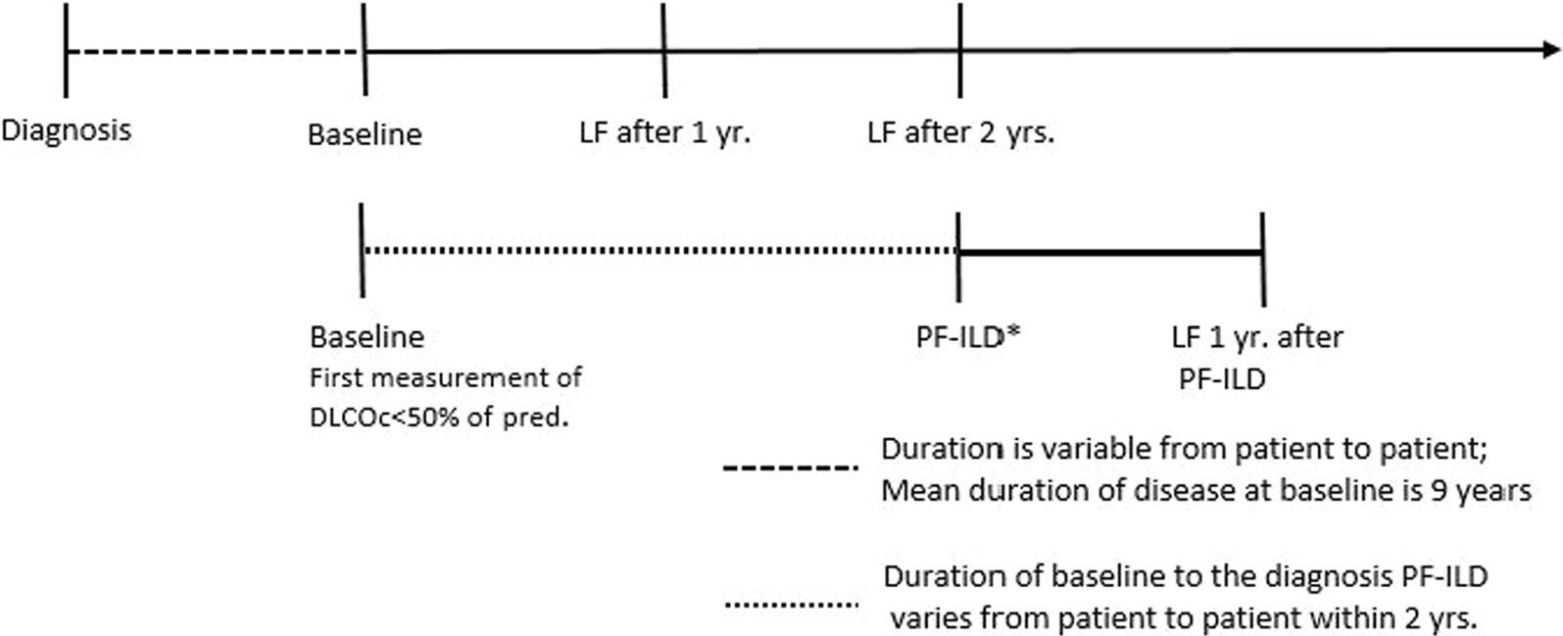
Study timeline. DLCOc = diffusion capacity for carbon monoxide corrected for haemoglobin; PF-ILD = progressive fibrosing interstitial lung disease. *The phenotype PF-ILD was established when the following conditions were met: > 10% fibrosis on the HRCT and progressive disease within 24 months of the baseline: a relative decline of FVC of 10% of predicted, a relative decline of FVC of 5–10% of predicted in combination with an increase of fibrosis or worsening of respiratory symptoms, or an increase of fibrosis on HRCT in combination with worsening of respiratory symptoms

At the baseline the following data were collected: the lung function, Scadding stage, organ involvement, HRCT data and treatment. Follow-up lung function data were collected 1 year and 2 years after the baseline. The following data were also collected: date of diagnosis, date of birth, sex and ethnicity. Research related lab work (e.g., MUC5B) was performed retrospectively from the ILD biobank. Figure 1 pictures the study timeline.

The composite score of Walsh was determined as described in the paper of Walsh et al. [9], and divides the population in patients with a good and poor prognosis. Patients with a CPI higher than 40 were classified as patients with poor prognosis. Patients with CPI ≤ 40, but a MPAD/AAD > 1 or extent of fibrosis > 20% were also classified as patients with a poor prognosis [9].

The survival period was calculated from baseline to the date of death or lung transplantation, or in the case of survivors to the last known contact. The mortality rate was calculated after 5 and after 10 years. Information concerning vital status of the patient and the cause of death was obtained from patient files or from the general practitioner. Transplant-free survival was defined as survival free of lung-transplantation or death. The study was approved by the Medical research Ethics Committees United (MEC-U) of the St. Antonius Hospital (R05-08A) and all subjects gave written informed consent.

### Lung function

The following lung function parameters were collected: forced vital capacity (FVC), forced expiratory volume in one second (FEV1), diffusion capacity for carbon monoxide corrected for haemoglobin (DLCOc). Finger-prick blood samples were used to estimate venous haemoglobin (Hb) prior to pulmonary function testing. The pulmonary function data were expressed in litres and as percentage of predicted (% pred.). Lung function data were collected from the moment of the first measurement of DLCOc < 50% of predicted (baseline) and after 2 years ± 3 months. In patients with PF-ILD we collected lung function data 1 year after the diagnosis PF-ILD. CPI at baseline was calculated as previously described by Wells and colleagues [13]: CPI = 91.0 − (0.65 × DLCO % of predicted) − (0.53 × FVC % of predicted) + (0.34 × FEV1% of predicted).

### High resolution computed tomography

High-resolution computed tomography (HRCT) was available in all patients at baseline ± 1 year, missing data were handled with pairwise deletion.

A thoracic radiologist with special expertise in ILD (DM) reviewed all HRCT’s according to the staging system described by Walsh et al. [9]. In short, the lungs were assessed for the extent of fibrosis, reticulation with or without honeycombing, groundglass and other patterns of disease (not defined as fibrosis or groundglass). The proportion of the total disease extent and the above mentioned three individual patterns were estimated to the nearest 5%. The radiologist described the absence or presence of traction bronchiectasis and emphysema.

Furthermore, the ratio between the diameter of the mean pulmonary artery and the diameter of the ascending aorta (MPAd/AAd ratio) was measured and subdivided into three categories [9]: (0) diameter of pulmonary trunk less than diameter of ascending aorta; (1) pulmonary trunk diameter/ascending aorta dimeter ratio greater than 1 but less than or equal to 1.25; (2) pulmonary trunk diameter/ascending aorta diameter ratio greater than 1.25.

In addition, HRCTs were screened for UIP-like (usual interstitial pneumonia like) pattern as described in the INPULSIS trial [14]. Because the inter-observer agreement for the criteria for UIP is moderate [15], two radiologists screened on a UIP-like pattern. In cases of disagreement a third radiologist made the consensus decision on the presence of UIP-like pattern. A UIP-like pattern was noted if a patient met criteria A and C, B and C or all three criteria A, B and C:

A. Definite honeycomb destruction with basal and peripheral predominance
B. Presence of reticular abnormality and traction bronchiectasis
C. Atypical features are absent, specifically nodules and consolidation. Ground glass opacity, if present, is less extensive than reticular pattern.

### MUC5B

Mucin 5B *(MUC5B)* promotor polymorphism was analysed as described before in the paper of van der Vis and colleagues [16]. In short, genomic deoxyribonucleic acid was extracted from peripheral blood of each individual using standard method. A pre‐designed taqman single nucleotide polymorphism genotyping assay and an ABI 7500Fast analyzer (Applied Biosystems, Foster City, CA) were used to genotype rs35705950. We compared the minor allele frequency of the patients of our cohort with the minor allele frequency of healthy control subjects. In the healthy control group 249 healthy unrelated Dutch Caucasians were included.

### PF-ILD

PF-ILD was defined as described in the INBUILD trial [8]. All patients with PF-ILD had more than 10% fibrosis on the HRCT. The disease was defined as progressive if the following criteria were met within 24 months of the baseline: a relative decline of FVC of 10% of the predicted value, a relative decline of FVC of 5–10% of the predicted value in combination with an increase of fibrosis or worsening of respiratory symptoms, or an increase of fibrosis on HRCT in combination with worsening of respiratory symptoms. Patients with incidental decrease of FVC (thus only one measurement of FVC loss followed by an increase of FVC) were not labelled as progressive disease. Our thoracic radiologist (DM) evaluated the HRCT for progression of fibrosis.

We collected the follow-up of lung function data from the moment that a patient met the criteria for PF-ILD was until 1 year thereafter. We have chosen the same follow-up duration of 1 year as was described in the large INBUILD trial.

### Statistics

Statistical analyses were performed using IBM SPSS version 24 and Graphpad prism software version 6.05. Continuous variables are expressed as mean ± standard deviation (parametric data) or median with interquartile range (non-parametric data). Differences between groups with continuous data were tested with student-t test or the Mann–Whitney U test where appropriate. Numeric data are expressed as number (percentage), and differences between non-continuous data were measures with chi-square test. Survival analysis was performed using the Kaplan–Meier curves. For the composite endpoint overall mortality patients who underwent lung transplantation were considered dead. Univariate Cox regression analysis was used to identify predictors for transplant-free survival. Subsequently, we used multivariate Cox regression analysis in order to demonstrate independent predictors of transplant-free survival. Candidate covariates from the univariate analysis were included for multivariate analysis if p < 0.05. No missing data substitutions were made. Missing data were handled with pairwise deletion.

## Results

The cohort consisted of 106 patients with advanced pulmonary sarcoidosis. The patient characteristics at baseline are outlined in Table 1. The mean age of this study cohort was 49 years and 68% of the patients were male. Median duration of disease prior to the first measurement of DLCO < 50% of predicted was 5 years. Mean CPI was 47 ± 9, and a CPI above 40 was measured in 87 patients. According to the algorithm of Walsh 98 patients (92%) of the patients are suspected to have a poor prognosis: 87 out of the 98 patients (89%) had a CPI above 40, 8 out of the 98 patients (8%) had more than 20% fibrosis on HRCT and 8 out of the 98 patients (8%) had a MPAd/AAd ratio of more than 1.

**Table 1:** Patient characteristics at first measurement of DLCO < 50% of predicted

		n = 106
Age		49 ± 13 years
Male/Female		72 (68)/34 (32)
White/ Non-white/Unknown		66 (62)/30 (28)/10 (9)
Histologic confirmation		104 (98)
Smoking history	Never	29 (27)
	Former	48 (45)
	Current	23 (22)
	Unknown	6 (6)
Cardiac involvement	Probable	6 (6)
	Possible	2 (2)
≥ 2 organs involved		95 (90)
Therapy	Corticosteroids	59 (56)
	Methotrexate	37 (35)
	Azathioprine	3 (3)
	Plaquenil	4 (4)
	Anti-TNF treatment	8 (8)
	None	26 (25)
	Unknown	4 (4)
Duration of disease prior to baseline time point		5 (13) years
Scadding stage	0	2 (2)
	I	4 (4)
	II	19 (18)
	III	12 (11)
	IV	63 (59)
	Unknown	6 (6)
PH	Diagnosed with RHC	9 (8)
	PH suspected on echocardiogram	3 (3)
FVC % predicted		70 ± 17
FEV1% predicted		60 ± 18
DLCOc% predicted		42 ± 7
CPI		47 ± 9
Walsh poor prognosis		98 (92)
*MUC5B* promotor polymorphism	GG	83 (78)
	GT	23 (22)
	TT	0

Data are shown as number (%) or mean ± SD, except for the duration of disease this is shown as median (IQR)

PH = pulmonary hypertension; FVC = forced vital capacity; FEV1 = forced expiratory volume in one second; DLCOc = diffusing capacity for carbon monoxide corrected for haemoglobin; RHC = right heart catheterization; CPI = composite physiologic index; *MUC5B* = Mucin 5B rs35705950

In addition, at baseline, 12 patients (11%) were diagnosed with PH based on mPAP > 25 mmHg during right heart catheterisation (n = 9) or compatible findings of transthoracic echocardiography (n = 3). The diagnosis of PH using echocardiography was based on a high tricuspid regurgitation maximum velocity of 4.4 m/s and secondary signs of pulmonary hypertension in one patient and in two patients based on secondary signs of pulmonary hypertension, such as right atrial dilatation, hypertrophy of right ventricle and paradoxical septal motion. In the patients who received right heart catheterisation mPAP (mean pulmonary arterial pressure) was 38 ± 13 mmHg. 76 patients (72%) were treated with immunosuppressive therapy at the baseline.

The minor allele frequency for *MUC5B* rs35705950 in our cohort was 11% and did not significantly differ from the minor allele frequency of 9% in the controls (p > 0.05).

### Change in lung function

Mean FVC at baseline was 70% of predicted and mean DLCO at baseline was 42% of predicted (Table 1).

After 2 years, four patients had died (4%) and FVC and DLCO were available for 59 and 51 patients, respectively. Evolution of lung function varied widely between patients. Change in FVC after 2 years follow-up ranged between − 34% and + 45% of predicted, whereas change in DLCO varied between − 11% and + 26%.

**Fig. 2:**
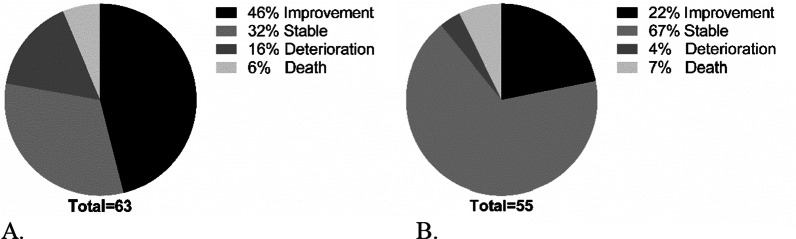
Proportions of patients with improved, stable or deteriorated lung function and patients who died. **A** improvement, stable or deteriorated FVC after 2 years; **B** improvement, stable or deteriorated DLCOc after 2 years. FVC: Improvement ≥ + 5% predicted; Stable − 5 to + 5% predicted; deterioration ≥ − 5% predicted. DLCOc: Improvement ≥ + 10% predicted; stable − 10 to + 10% predicted; deterioration ≥ − 10% predicted

After 2 years of follow-up 29 patients (46%) of the patients had improved FVC and 12 patients (22%) improved DLCOc (Fig. 2). Deterioration of FVC was in 10 (16%) of the patients, whereas only 2 (4%) had deterioration of DLCOc (Fig. 2).

### HRCT

HRCT data at baseline ± 1 year were available in 106 patients (Table 2). In this cohort, the median total disease extent of the lung was 70%. The most prominent HRCT pattern was groundglass, with a median of 21% involvement of the lungs. A total of 56 patients had more than 10% fibrosis on HRCT. Furthermore, only four (4%) patients had a UIP-like pattern on HRCT.

**Table 2:** HRCT characteristics at baseline in patients with a HRCT at baseline

HRCT characteristics		N = 106
Fibrosis	Presence of fibrosis	79 (75)
	> 10% of the lungs fibrosis	56 (53)
Total disease extent (%)		70; 30–90
Fibrosis; % of lung		12; 0–24
Groundglass; % of lung		21; 4–52
Other pattern; % of lung		7; 0–20
Traction bronchiectasis		59 (56)
Emphysema		22 (21)
MPAd/AAd category: 0/1/2		49 (46)/50 (47)/7 (7)
UIP-like pattern		4 (4)

Data are shown as number (percentage) or median; IQR

MPAd/AAd = mean pulmonary artery diameter/ascending aorta diameter; UIP = usual interstitial pneumonia

MPAd/AAd category (0) MPAd/AAd < 1; category (1) MPAd/AAd 1–1.25; category (2) MPAd/AAd > 1.25

### PF-ILD

In 94 patients we had enough data to apply the criteria for PF-ILD as used in the INBUILD trial. We found that 14 out of the 94 patients (15%) met the PF-ILD criteria.

**Fig. 3:**
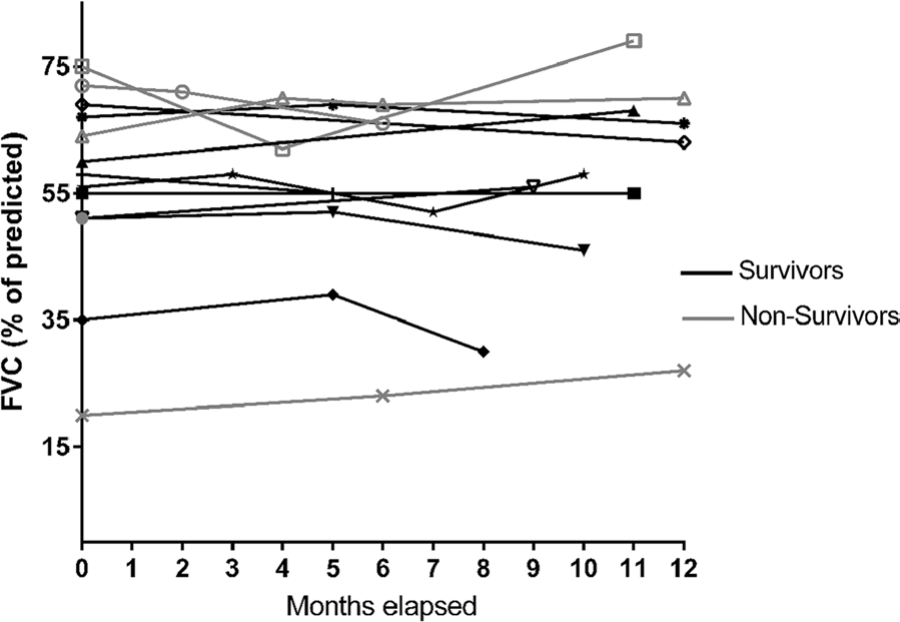
One year follow-up FVC (% of predicted) in patients with PF-ILD. Time 0 (T = 0) is the first time that patients fulfil the criteria for PF-ILD. No difference was found between survivors and non-survivors in change in FVC after 1 year follow-up in patients with PF-ILD (p > 0.05)

Lung function data after 1 year from the moment that patients had PF-ILD showed no significant increase or decrease (Fig. 3). In two patients follow-up lung function data were not available because they had already died. All patients received immunosuppressive therapy at the time they met the criteria of PF-ILD. Three patients switched immunosuppressive therapy within 1 year of follow-up.

Patients with PF-ILD are characterized by more fibrosis and traction bronchiectasis on HRCT (Table 3). Mortality in the PF-ILD group was significantly higher than in the non PF-ILD group (43% versus 13%; p = 0.013). One patient underwent a bilateral lung transplantation, 3 patients died because of end-stage fibrotic sarcoidosis and two patients died because of pneumonia in the PF-ILD group. There was no significant difference in *MUC5B* rs35705950 T-allele carriership between PF-ILD and non PF-ILD. However in the whole cohort (PF-ILD and non PF-ILD) three out of four patients with UIP pattern on HRCT carried the *MUC5B* T-allele.

**Table 3:** Characteristics of patients with PF-ILD versus non PF-ILD at baseline (DLCO < 50% of pred.)

		PF-ILD (n = 14)	Non PF-ILD (n = 80)	P-value
Age		52 ± 12	49 ± 13	NS
Male		10 (71)	51 (64)	NS
Ethnicity	White	7 (50)	51 (64)	NS
	Non-white	4 (29)	23 (29)	
	Unknown	3 (21)	6 (7)	
Smoking history	Never	5 (36)	20 (25)	NS
	Former	9 (64)	37 (46)	
	Current	0	18 (23)	
	Unknown	0	5 (6)	
CPI		48 ± 14	47 ± 8	NS
PH		4 (29)	8 (10)	NS
Total Disease extent		70; 48–90	73; 30–89	NS
% fibrosis		33; 18–37	8; 0–20	< 0.001
% groundglass		14; 5–25	25; 3–62	NS
% other pattern		12; 5–18	7; 0–24	NS
MPAd/AAd Ratio		1.0; 0.9–1.1	1.0; 0.9–1.1	NS
Emphysema		3 (21)	16 (20)	NS
Traction bronchiectasis		13 (93)	37 (46)	0.001
UIP like pattern		1 (7)	3 (4)	NS
*MUC5B;* rs35705950 carriers of T allele (genotype GT or TT)		3 (21)	18 (23)	NS
Deaths/lung transplantation		6 (43)	10 (13)	0.013

Age and CPI are shown as mean ± standard deviation. The rest of the table is shown as number (%) or median; IQR

CPI = composite physiological index; MPAd/AAd = mean pulmonary artery diameter/ascending aorta diameter; UIP = usual interstitial pneumonia; *MUC5B* = Mucin 5B; PH = Pulmonary hypertension

### Survival

#### Univariate analysis

A total of 15 patients died of whom in 14 patients it was definitely attributable to sarcoidosis. In one patient the cause of death was familial dilated cardiomyopathy (not sarcoidosis related). Three patients underwent bilateral lung transplantation, and four patients died on the waiting list.

In the total cohort, the 5- and 10-year overall mortality was 11% and 16%, respectively.

Univariate Cox regression analysis revealed four predictors for the composite endpoint of overall mortality (Table 4): CPI (HR 1.1; 95% CI 1.0–1.1; p = 0.049), PH (HR 3.8; 95% CI 1.0–14.3; p = 0.047), PF-ILD (HR 4.5; 95% CI 1.6–12.5; p = 0.004), and a UIP-like pattern on HRCT (HR 9.8; 95% CI 2.6–36.6; p = 0.001).

**Table 4:** Univariate and multivariate analysis of predictors of mortality and lung transplantation

		N	Univariate analysis for overall mortality and lung transplantation			Multivariate analysis for overall mortality and lung transplantation		
			HR	95% CI	P-value	HR	95% CI	P-value
Age		106	1.0	1.0–1.1	0.127			
Gender (male)		106	1.6	0.5–4.8	0.437			
Ethnicity: white		96	0.4	0.1–1.1	0.070			
Smoking	Ex	100	1.5	0.5–4.2	0.441			
	Current	100	5.0	0.6–38.2	0.124			
CPI		104	1.1	1.0–1.1	**0.049**	1.0	1.0–1.1	0.256
PH		106	3.8	1.0–14.3	**0.047**	4.6	1.1–20.3	**0.042**
PF-ILD		94	4.5	1.6–12.5	**0.004**	4.5	1.5–13.7	**0.008**
Scadding stage IV		100	1.5	0.5–4.0	0.451			
Total disease extension		106	1.0	1.0–1.0	0.438			
% Fibrosis		106	1.0	1.0–1.1	0.062			
% Groundglass		106	1.0	1.0–1.0	0.090			
% Other pattern		106	1.0	1.0–1.0	0.761			
MPA/AAD ratio		103	3.2	0.2–58.0	0.429			
Emphysema		106	1.0	0.3–3.1	0.987			
Traction bronchiectasis		106	1.3	0.5–3.3	0.627			
UIP like pattern		106	9.8	2.6–36.6	**0.001**	13.1	3.1–54.6	**< 0.001**
*MUC5B;* carriers of T allele		106	0.6	0.2–2.1	0.442			
Walsh poor prognosis		106	22.6	0.0–190	0.465			

HR = hazard ratio; 95% CI = 95% confidence interval; CPI = composite physiological index; PF-ILD = progressing fibrotic interstitial lung disease; MPAd/AAd = mean pulmonary artery diameter/ascending aorta diameter; UIP = usual interstitial pneumonia; *MUC5B* = Mucin5B; PH = pulmonary hypertension

#### Multivariate analysis

**Fig. 4:**
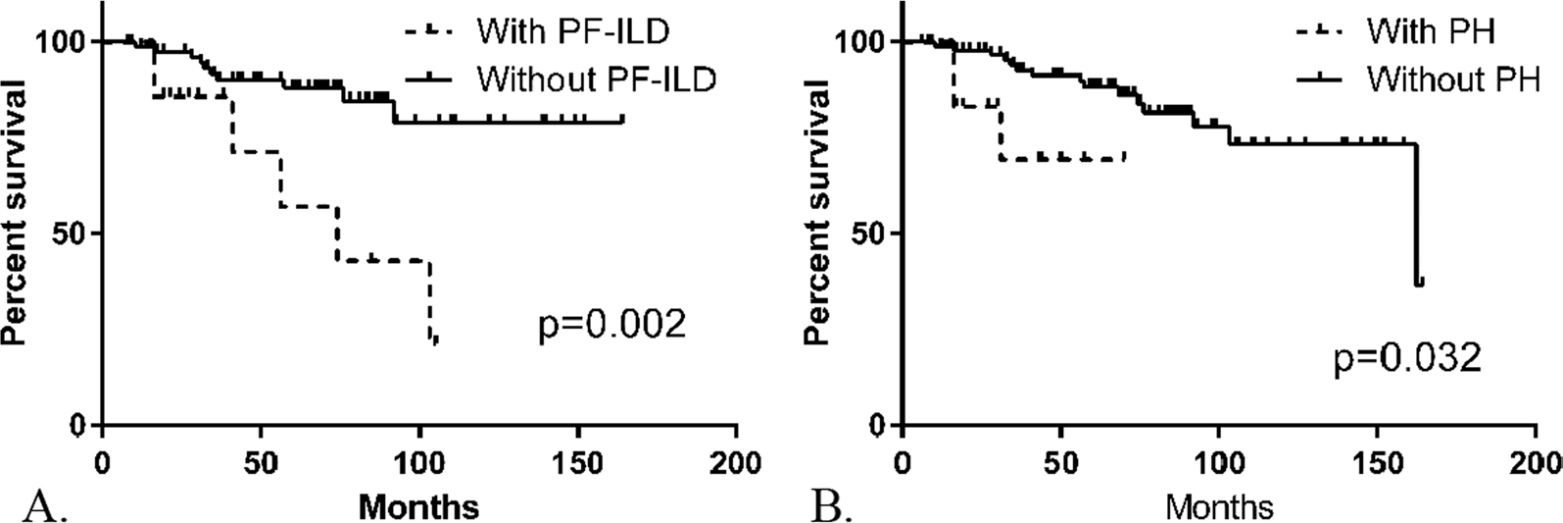
Kaplan Meier curves for the transplant-free survival. **A** Survival analysis in patients with and without PF-ILD; **B** survival analysis in patients with and without PH. Median survival patient with PF-ILD = 6 years; median survival patients with PH = 5 years

The following covariates were analysed with multivariate Cox regression analysis: CPI, PH, PF-ILD and UIP-like pattern. Multivariate Cox regression analysis demonstrated that three covariates are independent predictors of overall mortality and lung transplantation: PH, PF-ILD and UIP-like pattern (Table 4). Comparison of transplant-free survival in patients with and without PF-ILD is shown by Kaplan Meier curve in Fig. 4A and with and without PH in Fig. 4B.

## Discussion

In this study in patients with advanced pulmonary sarcoidosis based on a severely impaired diffusion capacity, we demonstrated that 15% of patients meet the current criteria for progressive fibrosing ILD (PF-ILD). In our study cohort, we found PH, PF-ILD and UIP-like pattern on HRCT to be independent predictors for all-cause mortality and lung transplantation. In the group of sarcoidosis who meet PF-ILD criteria, 43% died or underwent bilateral lung transplantation, whereas the overall mortality in the non-PF-ILD was 13%.

As no well-described definition of advanced pulmonary sarcoidosis exists, we chose to use DLCO as determining criterion. DLCO is reduced in 90% of the patients with advanced pulmonary sarcoidosis [17]. Furthermore, in the absence of fibrosis DLCO might be limited in patients with sarcoidosis, for example in patients with PH [18]. An important observation in our study is the significant heterogeneity in disease course. Although a severely impaired diffusion capacity in patients with IPF or CTD-ILD in general is associated with a poor prognosis [19], we see a remarkably high percentage of patients in our study cohort where FVC remains stable (32%) or even improved (46%) after 2 years of follow-up. One explanation could be that patients with sarcoidosis generally respond well to immunosuppressive therapy. In the observational multi-center study of Kampstra et al. (20) including 509 patients with different stages of sarcoidosis was found that 21% of the patients had an improvement of FVC and 17% had an improvement of DLCO after 2 years follow-up. The mean % of DLCO in our study cohort was 42% which is comparable with mean DLCO at baseline in, for example, the ASCEND, INPULSIS and INBUILD trial, 44%, 47% and 46% respectively [8, 21, 22].

We demonstrated that PF-ILD is rare, even in patients with advanced pulmonary sarcoidosis, as our data show that only 15% of our cohort fulfil the criteria of PF-ILD. In the overall sarcoidosis-population, the percentage of patients fulfilling the criteria of PF-ILD would be significantly lower because we used an inclusion criterion of at least one measurement of DLCOc < 50% predicted. A retrospective Italian study found that 41% of sarcoidosis patients with Scadding stage IV had PF-ILD, which is much higher than the prevalence what we found. This difference might be explained by the fact that in our study PF-ILD was diagnosed if patients had more than 10% fibrosis on HRCT and met the criteria for progressive disease according to the INBUIDL trial, whereas in the Italian study patients with Scadding stage IV who met the criteria for progressive disease were labelled as PF-ILD [23].

In the patients with PF-ILD we found a mean increase of 105 mL FVC after 1 year follow-up (data not shown), whereas in the placebo-group of the INBUILD trial a mean decrease of -187.8 mL after 1 year was found. However, our data cannot be compared with the outcomes of the INBUILD trial, because in the INBUILD trial tipping point analysis is used to impute FVC data in patients who died. Despite stable FVC after 1 year follow-up in patients with PF-ILD, our data also demonstrate that PF-ILD in patients with advanced pulmonary sarcoidosis is associated with a poor prognosis given the fact 43% of this group died from sarcoidosis related causes. From these findings, we might conclude that FVC does not give a realistic reflection of the severity of the disease. However, our lung function results should be interpreted with caution as the group composed only 14 patients. In the study of Behr and colleagues [24], describing the course of disease in IPF patients with and without antifibrotic therapy, was demonstrated that overall decline of FVC did not differ between patients with and without antifibrotic therapy, whereas they found a great significant difference in mortality between patients with and without antifibrotic therapy. CPI (incorporating FVC, FEV1 and DLCO) or 6-min walk test (6MWT) seem more suitable as clinical parameters to evaluate the therapeutic effect in sarcoidosis, as both parameters are clinical predictors of mortality in ILD [9, 25]. In the INBUILD trial, patients did not receive other therapy besides nintedanib or placebo, whereas in our cohort all patients with PF-ILD received immune suppressive treatment.

Nintedanib is not a curative treatment, and a relatively expensive drug [26] and can have substantial side-effects, and therefore patients and society can benefit from careful selection prior to initiation of this therapy. In the light of the recently published INBUILD trial, we suggest that patients with advanced pulmonary sarcoidosis fulfilling the criteria for PF-ILD might benefit from treatment with nintedanib.

Although the vast majority of the total cohort remained stable or even improved in terms of lung function, 12 patients (11%) eventually died or received lung transplantation within 5 years. Presence of PF-ILD, UIP-like pattern on HRCT and presence of PH were independent predictors of mortality. Unlike the findings in the United States and France [4, 6], we were not able to demonstrate racial or sex differences in mortality. However, this might be because the limited power of this study for mortality. Only a few studies focused on mortality in advanced pulmonary sarcoidosis [5, 6]. The overall 5-year mortality including lung transplantation was 11% in our study, which is similar to the results of Nardi and colleagues a 5-years mortality rate of 8.5% and that 4% underwent a lung transplantation [5] in a cohort with patients with only Scadding stage IV. Four out of six patients waiting for lung transplantation died. In a retrospective study, investigating mortality in patients with sarcoidosis on the transplant wait list, 18% died within 1 year and DLCO was found to be a strong predictor for death [27]. This study in combination with our results underlines the importance of appropriate timing for referral for lung transplantation.

Although 56 patients had more than 10% fibrosis on HRCT, a UIP-like pattern was found in only four patients. A UIP-like pattern is rare in sarcoidosis, therefore the question remains if patients with a UIP pattern in sarcoidosis might have two disorders or that a UIP pattern is a rare consequence of progressive fibrosis in sarcoidosis. The continuous inflammatory condition of the lungs may predispose to the development of a UIP in susceptible patients with sarcoidosis. In addition, pro-fibrotic genetic markers can play a role in the development of a UIP pattern in sarcoidosis. Three out of four patients with a UIP on HRCT carried the *MUC5B* T-allele. In RA-ILD it was shown that UIP is associated with presence of the *MUC5B* T-allele [28]. A similar situation may be present in sarcoidosis although UIP is much less frequent and subsequent studies focussing on this rare phenotype in sarcoidosis are needed to confirm this hypothesis. Thus far, only one other study has investigated the association between *MUC5B* variant and sarcoidosis. Stock et al. [29] demonstrated that *MUC5B* is not associated with fibrotic sarcoidosis (Scadding stage IV) nor with progression of sarcoidosis. However, they did not study the association between the presence of the UIP pattern in fibrotic sarcoidosis and *MUC5B* genotype.

The last independent predictor found for overall mortality was the presence of PH. It is well known that PH is associated with a poor prognosis in sarcoidosis [5–7]. In our cohort with advanced pulmonary sarcoidosis, 11% of the patients had PH at baseline. The incidence of PH in the overall sarcoidosis population varies widely from 3 to 20% [30–34], and in patients awaiting for lung transplantation the incidence is even higher varying from 39% up to 79% [27, 35]. In 58% of the patients with PH, fibrosis was found on HRCT. This is in line the study of Sulica et al. [36], this retrospective study found that 60% of the patients with PH had fibrosis.

As our study is retrospective, lung function and HRCT were not performed at strictly set times, and therefore we had some missing data. However, the aim of our study was to describe a cohort with advanced pulmonary sarcoidosis and we feel that our cohort is a realistic reflection of this phenotype in clinical practice.

## Conclusion

In conclusion, in this study on advanced pulmonary sarcoidosis defined on the basis of severely lowered DLCO (< 50%), a PF-ILD phenotype was found in 15%. Overall disease course showed remarkable heterogeneity. Although lung function in most patients improved or stabilised, overall 5-year and 10-year mortality in the whole cohort was substantial with 11% and 16%, respectively. Independent negative predictors of transplant-free survival were PH, UIP-like pattern and PF-ILD phenotype. Future research should focus on the efficacy and timing of antifibrotic therapy in patients with sarcoidosis with a PF-ILD phenotype.

## Data Availability

The datasets used and/or analysed during the current study are available from the corresponding author on reasonable request.

## Abbreviations

95% CI: 95% Confidence interval
CPI: Composite physiological index
CTD: Connective tissue disease
DLCO: Diffusion capacity of carbon monoxide
FEV1: Forced expiratory volume in one second
FVC: Forced vital capacity
HR: Hazard ratio
HRCT: High-resolution computed tomography
HP: Hypersensitivity pneumonitis
IPF: Idiopathic pulmonary fibrosis
MPAd/AAd: Mean pulmonary artery diameter/ascending aorta diameter
mPAP: Mean pulmonary arterial pressure
*MUC5B*: Mucin5B
PF-ILD: Progressing fibrotic interstitial lung disease
PH: Pulmonary hypertension
RHC: Right heart catheterization
UIP: Usual interstitial pneumonia
